# Hyperbaric oxygen reduces inflammation, oxygenates injured muscle, and regenerates skeletal muscle via macrophage and satellite cell activation

**DOI:** 10.1038/s41598-018-19670-x

**Published:** 2018-01-22

**Authors:** Takuya Oyaizu, Mitsuhiro Enomoto, Naoki Yamamoto, Kunikazu Tsuji, Masaki Horie, Takeshi Muneta, Ichiro Sekiya, Atsushi Okawa, Kazuyoshi Yagishita

**Affiliations:** 1 Department of Orthopaedic Surgery, Tokyo Medical and Dental University, Bunkyo-ku, Tokyo, 113-8519 Japan; 2 Hyperbaric Medical Center, Medical Hospital, Tokyo Medical and Dental University, Tokyo, 113-8519 Japan; 3 Sports Medicine Clinical Center, Medical Hospital, Tokyo Medical and Dental University, Bunkyo-ku, Tokyo, 113-8519 Japan; 4 Department of Cartilage Regeneration, Tokyo Medical and Dental University, Bunkyo-ku, Tokyo, 113-8519 Japan; 5 Department of Joint Surgery and Sports Medicine, Tokyo Medical and Dental University, Bunkyo-ku, Tokyo, 113-8519 Japan; 6 Center for Stem Cell and Regenerative Medicine, Tokyo Medical and Dental University, Bunkyo-ku, Tokyo, 113-8519 Japan

## Abstract

Hyperbaric oxygen treatment (HBO) promotes rapid recovery from soft tissue injuries. However, the healing mechanism is unclear. Here we assessed the effects of HBO on contused calf muscles in a rat skeletal muscle injury model. An experimental HBO chamber was developed and rats were treated with 100% oxygen, 2.5 atmospheres absolute for 2 h/day after injury. HBO reduced early lower limb volume and muscle wet weight in contused muscles, and promoted muscle isometric strength 7 days after injury. HBO suppressed the elevation of circulating macrophages in the acute phase and then accelerated macrophage invasion into the contused muscle. This environment also increased the number of proliferating and differentiating satellite cells and the amount of regenerated muscle fibers. In the early phase after injury, HBO stimulated the IL-6/STAT3 pathway in contused muscles. Our results demonstrate that HBO has a dual role in decreasing inflammation and accelerating myogenesis in muscle contusion injuries.

## Introduction

Among soft tissue injuries, muscle contusion injury is the most common sport-related injury, not only for athletes but also for recreational players. Such injuries may cause acute and/or chronic pain or disruption of innate muscle function, which can result in an inability to return to competition or to daily life activities^1,2^. Early and complete recovery from injury is one of the greatest concerns. Thus, treatment options for muscle injury such as massage, cryotherapy, and hyperbaric oxygen treatment (HBO) are being considered for use in clinical practice. Among these treatments, HBO has been proposed to have the highest potential as an effective adjunct treatment for muscle recovery^3–5^. However, the mechanism by which HBO could facilitate healing after skeletal muscle injury has not been established, and HBO is not yet recognized as a clinical treatment for skeletal muscle injury. Identification of the mechanisms underlying the potential therapeutic effects of HBO is essential for establishing HBO as a new treatment for muscle injury.

HBO consists of breathing pure oxygen at a high atmospheric pressure; the standard pressure is 2.0–2.8 atmospheres absolute (ATA) for 60–90 min generated by pressurized air or oxygen inside the chamber. HBO has two effects: oxygen delivery that is suitable for treating ischemic diseases and infection, and a compression effect that is essential for treating decompression illness. Based on these effects, HBO is indicated for various diseases such as carbon monoxide poisoning, osteomyelitis, decompression illness, and acute cerebral edema^6,7^. Recently, experimental HBO studies have been reported for muscular injury, but the injury models used, such as drug-induced injury, did not recapitulate the clinical condition^8–12^. It is therefore necessary to develop an animal skeletal muscle injury model for mechanistic evaluation of treatments such as HBO applied to muscle injuries in contact sports. In this study, we developed a stable muscle contusion injury model in rats based on the mass-drop method, and evaluated how the hyperbaric and hyper-oxygenated environment affected oxygenation, inflammation and hindlimb swelling as acute changes, as well as myofiber regeneration as a subacute change, after injury.

## Results

### HBO improves the hypoxic environment immediately and maintains high oxygenation in contused muscle

**Figure 1:**
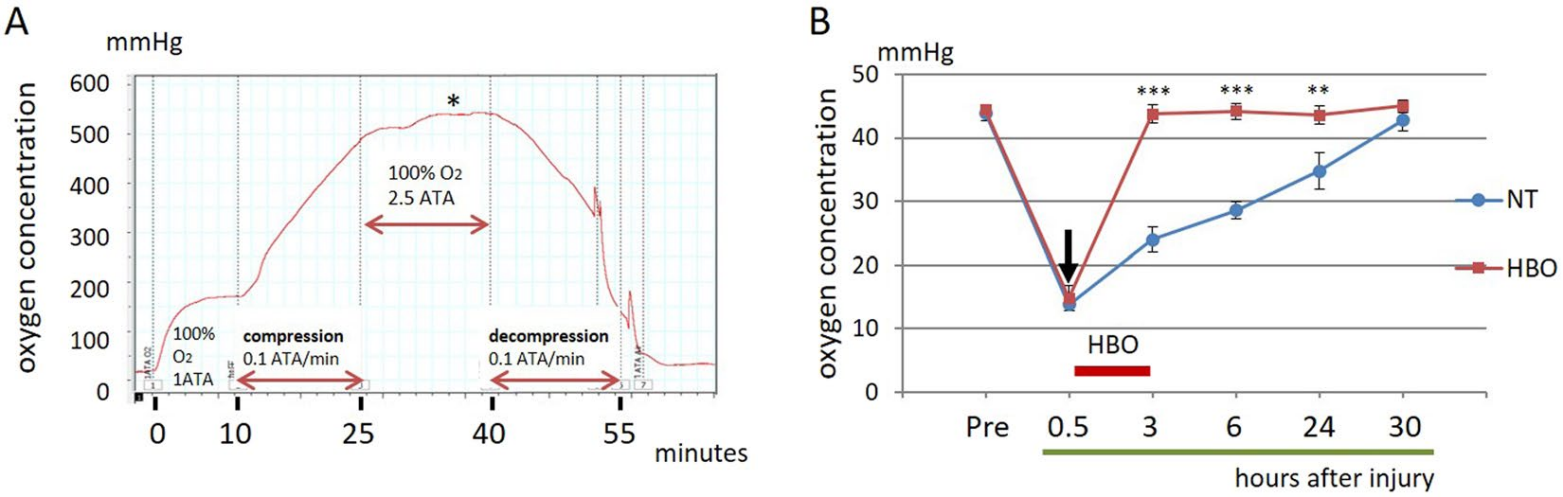
HBO reduced the hypoxic environment and maintained high oxygenation in contused muscle. (**A**) O_2_ concentration monitoring using a needle-type probe shows real-time oxygenation in the contused muscle during HBO. The oxygen concentration increased to 540 mmHg (asterisk) during HBO in the experimental protocol (100% oxygen for 15 minutes). Artifacts from body motion were observed during decompression. (**B**) O_2_ concentration in the injured muscle. The tissue oxygen concentration decreased from 45 to 15 mmHg within 30 minutes after injury (arrow). After one session of HBO, hypoxia was reduced and oxygenation was maintained for 30 hours. n = 5. ***P < 0.001, **P < 0.01 using two-way ANOVA followed by Bonferroni post-tests. Data are the mean ± SEM.

The oxygen concentration in the calf muscles was physiologically evaluated from before injury to 30 hours after injury in the experimental HBO chamber. The total experimental time was 60 minutes. The oxygen concentration increased to 540 mmHg during HBO and decreased to 45 mmHg at the end of HBO (Fig. 1A). Under the condition of 1.0 ATA with air, the tissue oxygen concentration decreased from 45 to 15 mmHg within 30 minutes after injury (pre-contusion: NT vs. HBO, 44.0 ± 0.56 mmHg vs. 44.4 ± 0.27 mmHg; 30 minutes: NT vs. HBO, 13.8 ± 0.41 mmHg vs. 14.8 ± 0.87 mmHg). Thirty hours were required for the concentration to recover to pre-contusion levels in the non-treatment (NT) group without HBO. After one session of HBO, the hypoxia was significantly improved at 3 hours (P < 0.001), 6 hours (P < 0.001) and 24 hours (P < 0.01) (3 hours: NT vs. HBO, 24.0 ± 0.88 mmHg vs. 43.8 ± 0.63 mmHg; 6 hours: NT vs. HBO, 28.6 ± 0.61 mmHg vs. 44.2 ± 0.57 mmHg; 24 hours: NT vs. HBO, 34.8 ± 1.27 mmHg vs. 43.6 ± 0.63 mmHg) (Fig. 1B). Oxygenation was maintained for 30 hours in the contused muscle. HBO thus resulted in abundant oxygenation of injury-induced hypoxic skeletal muscle.

### HBO reduces hindlimb volume and muscle wet weight after contusion injury

**Figure 2:**
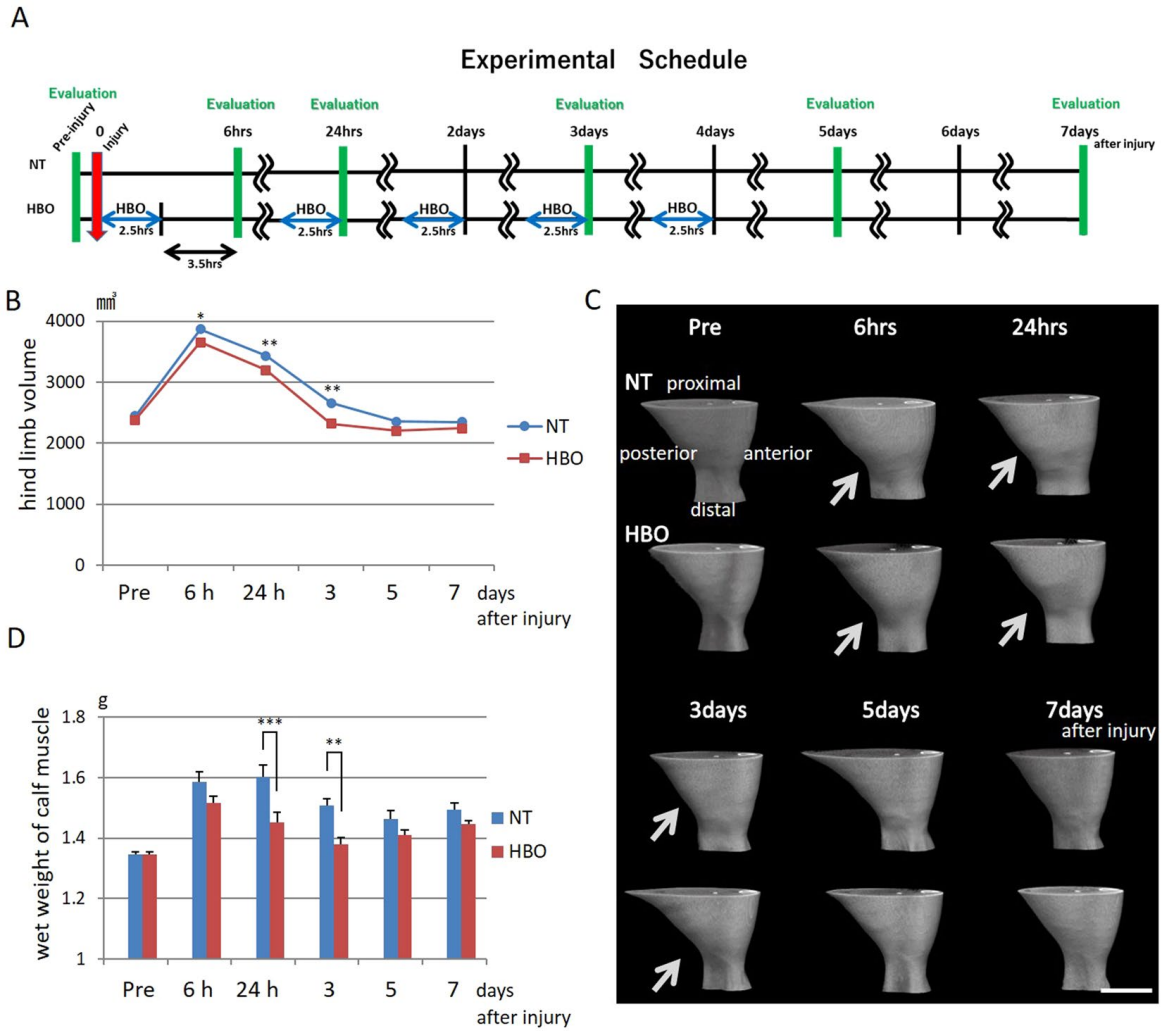
HBO reduced the volume of the injured hindlimb and muscle wet weight. (**A**) Treatment and evaluation schedule of hindlimb volume and calf muscle wet weight. (**B**) µCT evaluation of hindlimb volume after injury. The volume in the HBO group was lower than that in the NT group at 6 hours, 24 hours, and 3 days after injury. n = 13–20. **P < 0.01, *P < 0.05 using two-way ANOVA followed by Bonferroni post-tests. (**C**) 3D images of hindlimbs. Reduced swelling (arrows) was observed in the HBO group. Scale bar: 10 mm. (**D**) Wet weight of the calf muscles. The wet weight in the HBO group was lower than that in the NT group at 24 hours and 3 days after injury. n = 8. ***P < 0.001, **P < 0.01 using two-way ANOVA followed by Bonferroni post-tests. Data are the mean ± SEM.

Rats received non-treatment or repeated HBO sessions after injury. The treatment and evaluation schedule of hindlimb volume and calf muscle wet weight are described in Fig. 2A. The changes in injured hindlimb volume over time were evaluated before and 6 hours, 24 hours, 3 days, 5 days, and 7 days after injury in the NT and HBO groups by µCT (Fig. 2B). The volume immediately increased at 6 hours after injury, and then gradually decreased until 5 days after injury. At 6 hours (P < 0.01), 24 hours (P < 0.01) and 3 days (P < 0.05) after injury, the volume in the HBO group was significantly lower than that in the NT group (6 hours: NT vs. HBO, 4051 ± 21 mm^3^ vs. 3820 ± 21 mm^3^, 24 hours: NT vs. HBO, 3588 ± 13 mm^3^ vs. 3310 ± 13 mm^3^; day 3: 2775 ± 13 mm^3^ vs. 2459 ± 28 mm^3^). The 3D reconstruction images showed that the distal calf was thinner in the HBO group than in the NT group at 6 hours, 24 hours and 3 days after injury (Fig. 2C).

The wet weight of the isolated calf muscles was measured before and 6 hours, 24 hours, 3 days, 5 days, and 7 days after injury. The wet weight peaked at 6 hours in the HBO group and 24 hours in the NT group, and the values were significantly lower in the HBO group than in the NT group at 24 hours (P < 0.001) (NT vs. HBO, 1.60 ± 0.04 g vs. 1.45 ± 0.03 g) and 3 days (P < 0.01) (NT vs. HBO, 1.51 ± 0.02 g vs. 1.38 ± 0.02 g) after injury (Fig. 2D). HBO thus resulted in a lower limb volume and wet weight of the calf muscle after contusion-induced acute swelling.

### HBO decreases the extracellular space and vascular permeability, and repeated HBOs increase the number of newly regenerating myofibers

**Figure 3:**
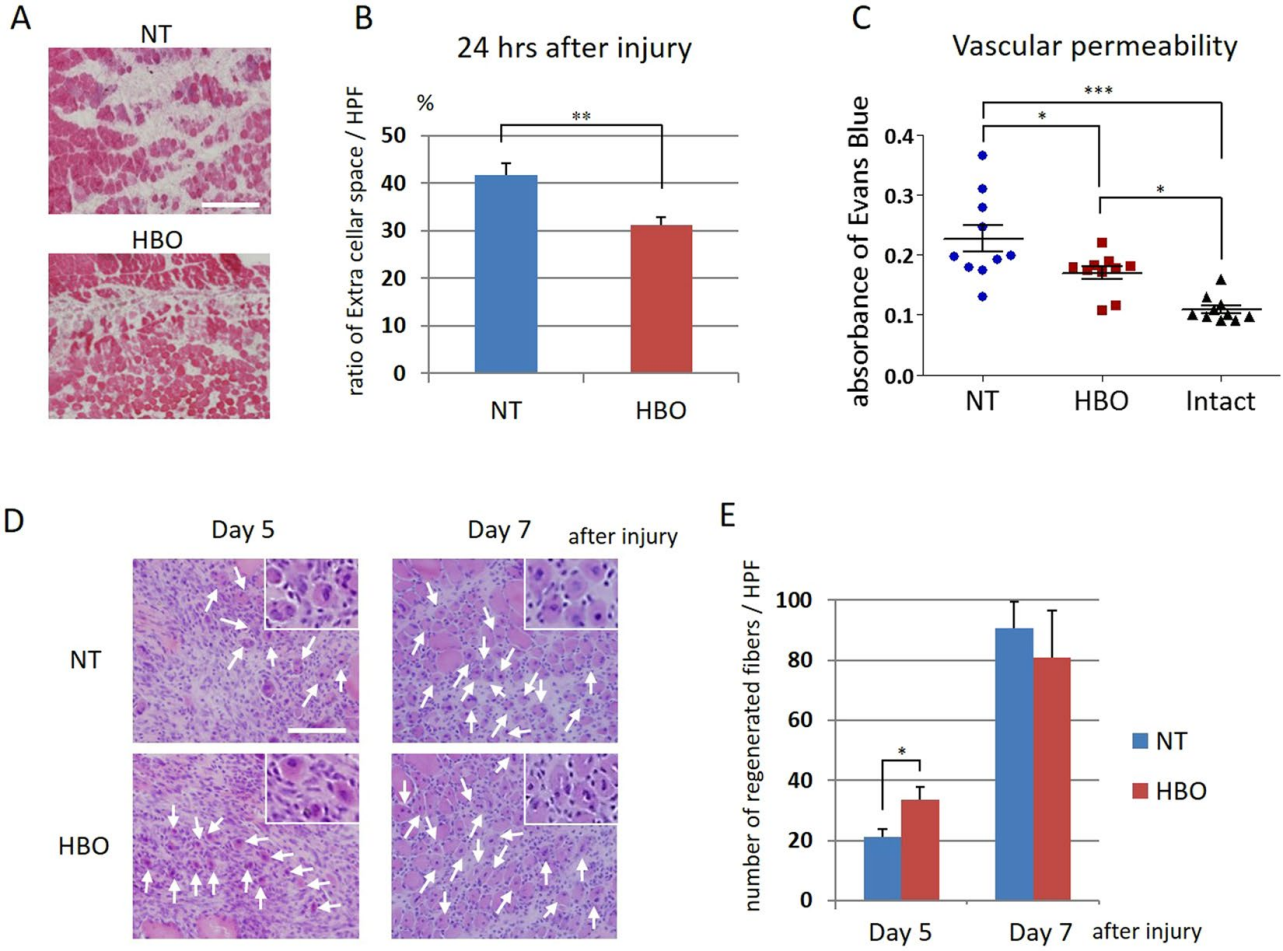
HBO improved the early post-injury muscle environment and increased myofiber regeneration. (**A**) Representative image of the extracellular space (non-stained area) in H&E-stained sections. Scale bar: 100 µm (**B**) The ratio of the extracellular space per HPF was lower in the HBO group. n = 5. **P < 0.01 using Welch’s test. (**C**) The absorbance of Evans Blue in the supernatant from homogenized muscles was higher in the NT and HBO groups than in the intact group. The absorbance in the HBO group was lower than that in the NT group. n = 10. ***P < 0.001, *P < 0.05 using one-way ANOVA followed by Bonferroni post-tests. (**D**) Nucleus-centered myofibers (insets) were observed from 5 days after injury in the NT group. Regenerated myofibers (arrows) were counted. Scale bars: 100 µm (**E**) The number of regenerating myofibers in the HBO group was higher than that in the NT group at 5 days. n = 5. *P < 0.05 using Student’s t-test. Data are the mean ± SEM.

We investigated whether any histological changes occurred in injured muscles at 24 hours after HBO. The extracellular spaces were larger in the NT group than in the HBO group at 24 hours after injury (Fig. 3A). The proportion of extracellular space in the high-power field (HPF) containing the injury site was significantly lower in the HBO group than in the NT group at 24 hours after injury (P < 0.01) (NT, 41.6 ± 2.6%; HBO, 31.2 ± 1.7%) (Fig. 3B). Vascular permeability was also evaluated based on Evans Blue leakage from the vasculature into the muscle. The absorbance values of Evans Blue in the supernatant from homogenized muscles were significantly higher in the NT (P < 0.001) and HBO (P < 0.05) groups than in the intact group. The absorbance values in the HBO group were significantly lower than those in the NT group (P < 0.05) (intact, 0.11 ± 0.02; NT, 0.23 ± 0.07; HBO, 0.17 ± 0.03) (Fig. 3C).

Repeated HBOs were performed and histological changes were chronologically analyzed in the injured muscles until 7 days after injury. In this model, newly regenerated myofibers with central nuclei began to appear at 5 days, and their numbers then increased by 7 days after injury (Figure S1). More regenerating fibers were observed in the HBO group than in the NT group at 5 days after injury (Fig. 3D). Quantitative analyses showed that the number of regenerating myofibers was significantly higher in the HBO group than in the NT group at 5 days (P < 0.05) (NT, 21.2 ± 2.58 fibers/HPF; HBO, 33.75 ± 4.03 fibers/HPF) after injury (Fig. 3E).

### HBO increases the maximum force-producing capacity in twitch and tetanic contraction of injured muscle

**Figure 4:**
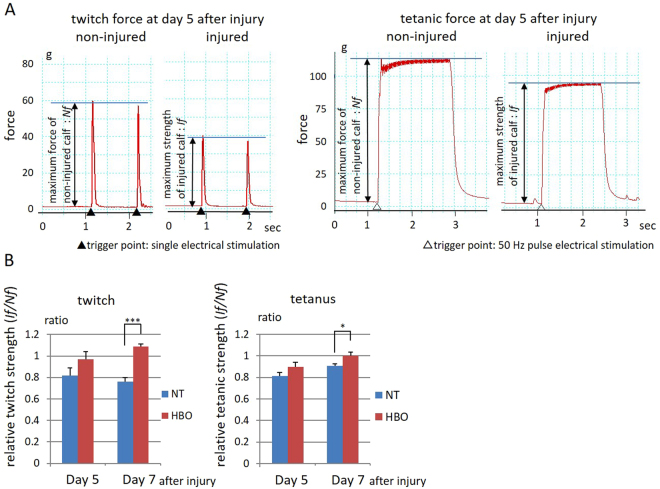
HBO increased the isometric tensile strength of the gastrocnemius muscle. (**A**) Twitch and tetanic forces were recorded and ratios of injured leg (*If*) to non-injured leg forces (*Nf*) were calculated. (**B**) The ratio of twitch muscle strength in the HBO group was higher than that in the NT group at 7 days. The maximum strength of tetanic forces in the HBO group was higher than that in the NT group at 7 days. n = 6. ***P < 0.001, *P < 0.05 using Student’s t test. Data are the mean ± SEM.

In a functional analysis, we measured the muscle isometric tensile strength of the injured muscle at days 5 and 7 after injury. The ratios of the twitch and tetanic muscle isometric tensile strength of the injured (right) to non-injured (left) legs in the same rats were evaluated (Fig. 4A). The twitch muscle strength in the HBO group was stronger than that in the NT group at 5 days (NT, 0.82 ± 0.07; HBO, 0.97 ± 0.07), and significantly stronger than that in the NT group at 7 days (P < 0.001) (NT, 0.76 ± 0.04; HBO, 1.09 ± 0.02) (left panel in Fig. 4B). The tetanic muscle isometric tensile strength in the HBO group was stronger than that in the NT group at 5 days (NT, 0.81 ± 0.03; HBO, 0.90 ± 0.04), and significantly stronger than that in the NT group at 7 days (P < 0.05) (NT, 0.91 ± 0.02; HBO, 1.00 ± 0.03) (right panel in Fig. 4B). Repeated HBOs thus advanced the recovery of twitch and tetanic muscle isometric strength.

### HBO changes the proportions of circulating and accumulating CD11b- and CD68-positive cells and accumulating CD163- and CD206-positive cells in injured muscle

**Figure 5:**
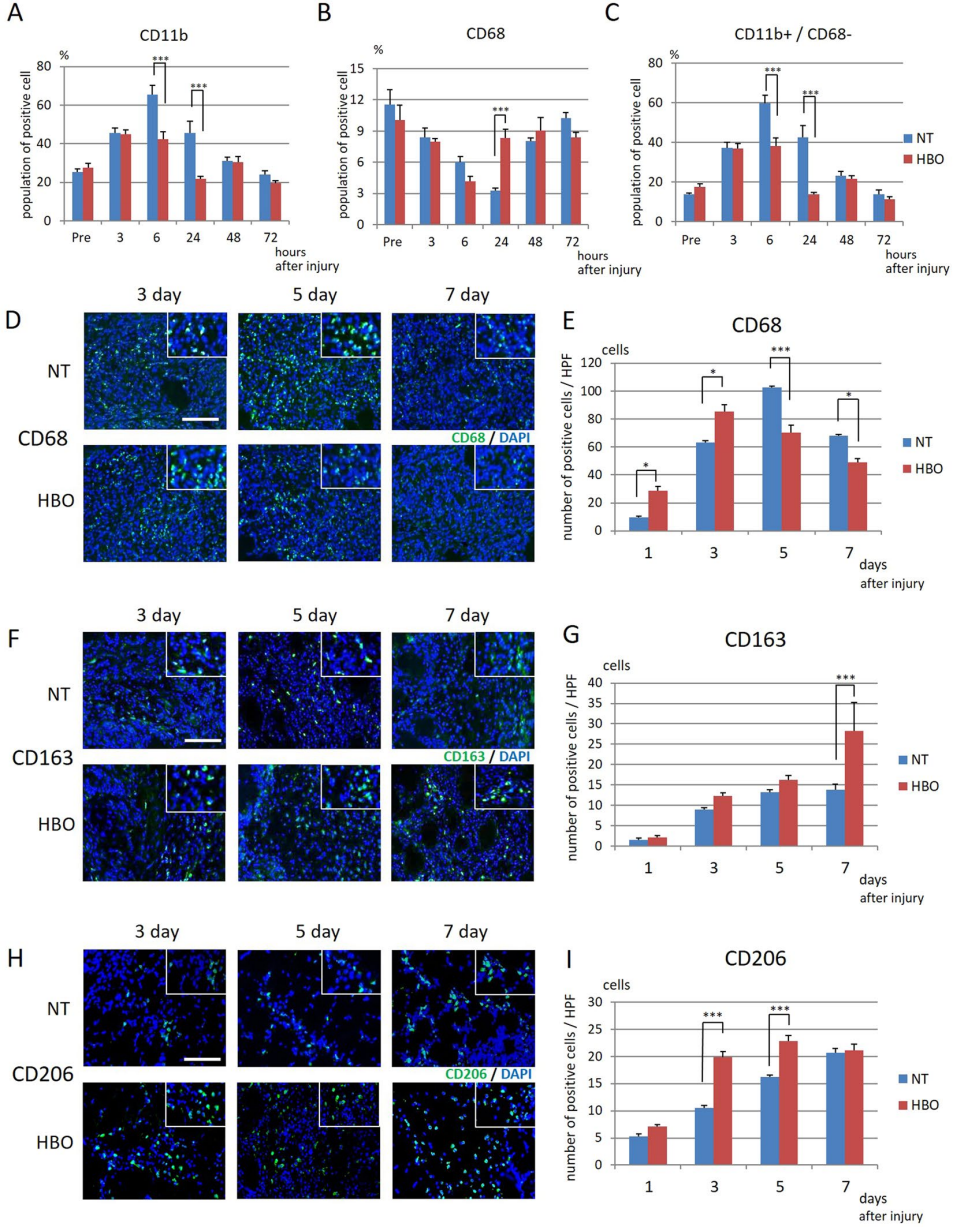
HBO decreased the amount of early circulating macrophages and facilitated macrophage infiltration in injured muscles. (**A**) The proportion of CD11b-positive cells was reduced in the HBO group at 6 hours and 24 hours. (**B**) The proportion of CD68-positive cells was increased in the NT group at 6 hours and in the HBO group at 24 hours. (**C**) The proportion of CD11b-positive/CD68-negative cells was lower in the HBO group than in the NT group at 6 hours and 24 hours, n = 5. ***P < 0.001 using two-way ANOVA followed by Bonferroni post-tests. (**D**) Representative images of CD68-positive cells colocalized with DAPI (insets). (**E**) The number of CD68-positive cells was increased in the HBO group at 1 day and 3 days after injury. The number of CD68-positive cells was increased in the NT group at 5 days and 7 days after injury, n = 5. ***P < 0.001, *P < 0.05 using two-way ANOVA followed by Bonferroni post-tests. (**F**) Representative image of CD163-positive cells. Scale bar: 100 µm. (**G**) The number of CD163-positive cells colocalized with DAPI (insets) in the HBO group was increased at 7 days, n = 5. ***P < 0.001 using two-way ANOVA followed by Bonferroni post-tests. (**H**) Representative image of CD206-positive cells. Scale bar: 100 µm. (**I**) The number of CD206-positive cells colocalized with DAPI (insets) in the HBO group was increased at 3 days and 5 days, n = 5. ***P < 0.001 using two-way ANOVA followed by Bonferroni post-tests. Data are the mean ± SEM.

We investigated whether the proportions of circulating CD11b- (inflammatory cell marker) and CD68- (monocyte marker) positive cells changed in the arterial blood within 72 hours after HBO. The proportion of CD11b-positive cells was significantly higher in the NT group than in the HBO group at 6 hours (P < 0.001) (NT, 65.5 ± 4.6%; HBO, 42.2 ± 4.1%) and 24 hours (P < 0.001) (NT, 45.6 ± 6.1%; HBO, 21.9 ± 1.1%) after injury (Fig. 5A). The proportion of CD68-positive cells was significantly higher in the HBO group than in the NT group at 24 hours (P < 0.001) (NT, 3.3 ± 0.2%; HBO, 8.3 ± 0.88%) (Fig. 5B). The proportion of CD11b-positive cells after subtraction of CD68-positive cells was significantly lower in the HBO group than in the NT group at 6 hours (P < 0.001) (NT, 59.5 ± 4.2%; HBO, 38.1 ± 4.1%) and 24 hours (P < 0.001) (NT, 42.3 ± 6.2%; HBO, 13.6 ± 1.2%) after injury (Fig. 5C). HBO reduced the proportion of circulating inflammatory cells and induced recovery of circulating monocytes, which were then expected to differentiate into macrophages in the injured tissue.

We also investigated whether macrophage accumulation in injured muscles changed by 7 days after HBO. CD68-positive cells were observed at the injury site from 1 day after injury. At 3 days, a higher number of CD68-positive cells was observed in the HBO group than in the NT group, whereas at 5 and 7 days, more CD68-positive cells were observed in the NT group (Fig. 5D). Additionally, the number of CD68-positive cells in the HBO group was significantly increased at 1 day (P < 0.05) (NT, 9.7 ± 2.5/HPF; HBO, 28.5 ± 3.3/HPF) and 3 days (P < 0.05) after injury (NT, 63.4 ± 4.6/HPF; HBO, 85.3 ± 4.7/HPF), and then significantly decreased at 5 days (P < 0.001) (NT, 102.7 ± 10.0/HPF; HBO, 70.3 ± 5.5/HPF) and 7 days (P < 0.05) after injury (NT, 68.0 ± 3.3/HPF; HBO, 48.9 ± 2.7/HPF) (Fig. 5E). The peak infiltration of CD68-positive cells into the injury site occurred 2 days earlier in the HBO group than in the NT group.

CD163-positive cells and CD206-positive cells (both anti-inflammatory macrophage markers) were observed around the injury site from 3 days after injury (Fig. 5F,H). Furthermore, the number of CD163-positive cells in the HBO group was significantly increased at 7 days (P < 0.01) (CD 163: NT, 13.8 ± 1.3/HPF; HBO, 28.2 ± 7.0/HPF) after injury (Fig. 5G). The number of CD206-positive cells in the HBO group was significantly increased at 3 days (P < 0.001) (CD 206: NT, 10.6 ± 1.0/HPF; HBO, 19.8 ± 1.1/HPF) and 5 days (P < 0.001) (CD 206: NT, 16.2 ± 0.4/HPF; HBO, 22.8 ± 1.0/HPF) after injury (Fig. 5I). Remarkable infiltration of CD206-positive cells occurred in injured muscles 3 days after injury, especially in the HBO group. HBO thus accelerated the peak of pan-macrophage infiltration and induced higher infiltration of anti-inflammatory macrophages in the injured muscles.

### HBO changes the numbers of Pax7- and MyoD-positive cells and increases the number of Pax7-positive cells colocalized with Ki67 in injured muscle

**Figure 6:**
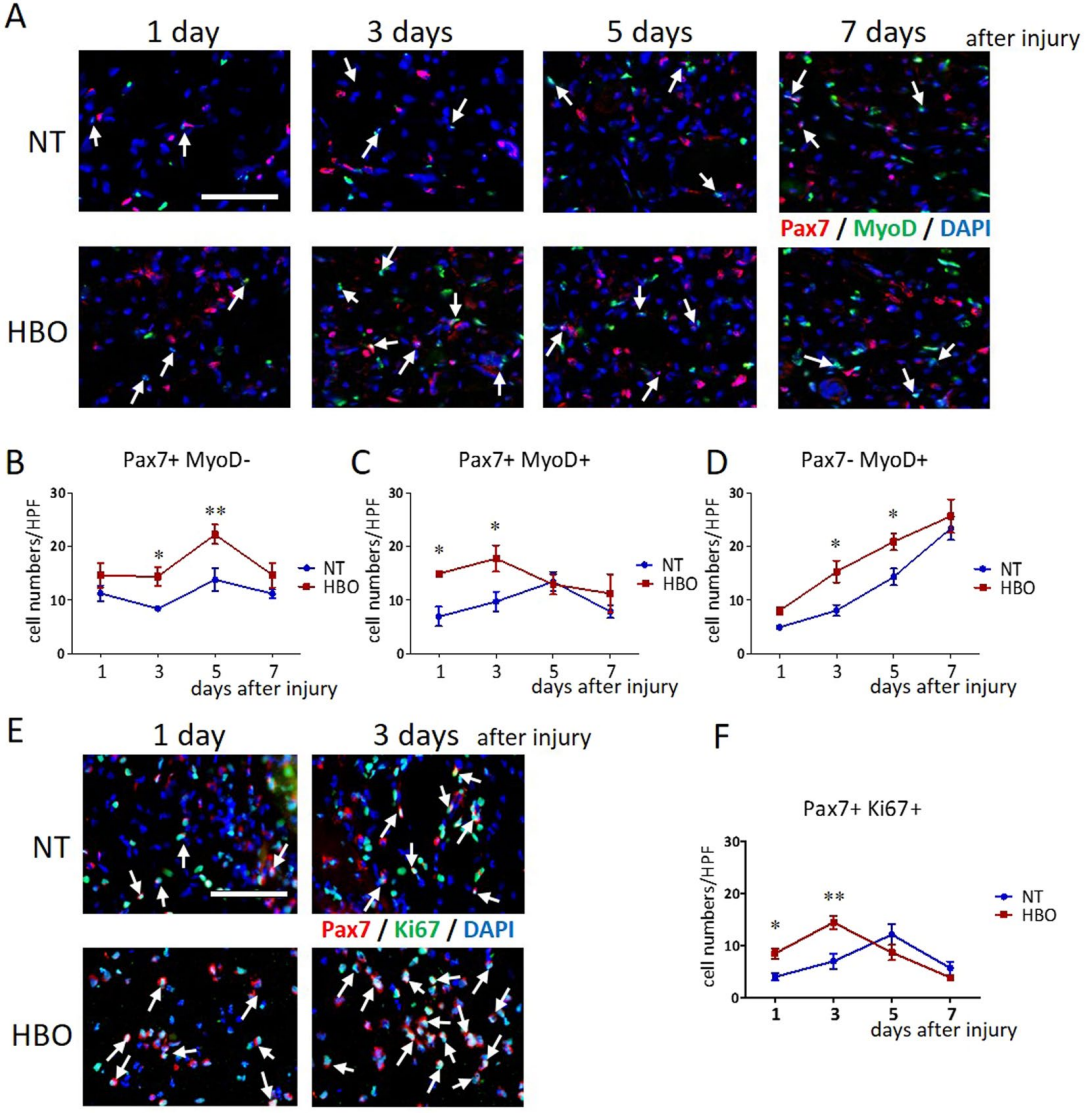
HBO accelerated the proliferation and differentiation of satellite cells in injured muscle. (**A**) Merged images, cells positive for Pax7 (red), MyoD (green), and DAPI (blue) in injured muscle (arrows). Scale bar: 50 µm. (**B**) The number of Pax7 + /MyoD- cells was significantly increased in the HBO group at 3 days and 5 days. (**C**) The number of Pax7^+^/MyoD^+^ cells was increased in the HBO group at 1 day and 3 days. (**D**) The number of Pax7^−^/MyoD^+^ cells were increased in the HBO group at 3 days and 5 days, n = 5. **P < 0.01, *P < 0.05 using two-way ANOVA followed by Bonferroni post-tests. (**E**) Merged images, cells positive for Pax7 (red), Ki67 (green), and DAPI (blue) in injured muscle (arrows). (**F**) The number of Pax7^+^/Ki67^+^ cells was increased in the HBO group at 1 day and 3 days, and peaked at 3 days in the HBO group and at 5 days in the NT group, n = 5. *P < 0.05 using two-way ANOVA followed by Bonferroni post-tests. Data are the mean ± SEM.

We investigated whether the proliferation and differentiation of satellite cells changed in injured muscles after HBO. Pax7- and MyoD-positive cells were observed at 1 day to 7 days after injury (Fig. 6A). The number of Pax7^+^/MyoD^−^ cells (quiescent satellite cells) in the injured muscle was significantly higher in the HBO group at 3 days (P < 0.01) (NT, 8.4 ± 0.5/HPF; HBO, 14.4 ± 1.7/HPF) and 5 days (P < 0.01) (NT, 13.8 ± 2.1/HPF; HBO, 22.3 ± 1.8/HPF) than in the NT group (Fig. 6B). The number of Pax7^+^/MyoD^+^ cells (proliferating satellite cells) was significantly higher in the HBO group than in the NT group at 1 day (P < 0.05) (NT, 7.0 ± 1.8/HPF; HBO, 14.9 ± 1.2/HPF) and 3 days (P < 0.05) (NT, 9.7 ± 1.8/HPF; HBO, 17.8 ± 2.47/HPF) (Fig. 6C). The number of Pax7^−^/MyoD^+^ cells (differentiated satellite cells) was significantly higher in the HBO group than in the NT group at 3 days (P < 0.05) (NT, 8.1 ± 1.0/HPF; HBO, 15.3 ± 2.0/HPF) and 5 days (P < 0.05) (NT, 14.4 ± 1.6/HPF; HBO, 20.9 ± 1.6/HPF) (Fig. 6D). The number of Pax7-positive cells peaked at day 5, and the number of MyoD-positive cells gradually increased until day 7. HBO accelerated satellite cell proliferation and differentiation, and then increased the number of quiescent and differentiated satellite cells.

For further evaluation of proliferating cells, we investigated the number of Pax7-positive cells also positive for the proliferation marker Ki67 (Pax7^+^/Ki67^+^ cells) in injured muscles (Fig. 6E). A higher number of Pax7^+^/Ki67^+^ cells was present in the HBO group than in the NT group at 1 day (NT, 4.0 ± 0.7/HPF; HBO, 8.5 ± 1.0/HPF) and 3 days (NT, 7.0 ± 1.5/HPF; HBO, 14.5 ± 1.3/HPF) after injury (Fig. 6F). The peak number of Pax7^+^/Ki67^+^ cells occurred at 3 days in the HBO group and 5 days in the NT group. HBO thus activated early proliferation of satellite cells in the acute phase after injury.

### HBO activates the IL-6/STAT3 pathway in injured muscle in the acute phase after injury

**Figure 7:**
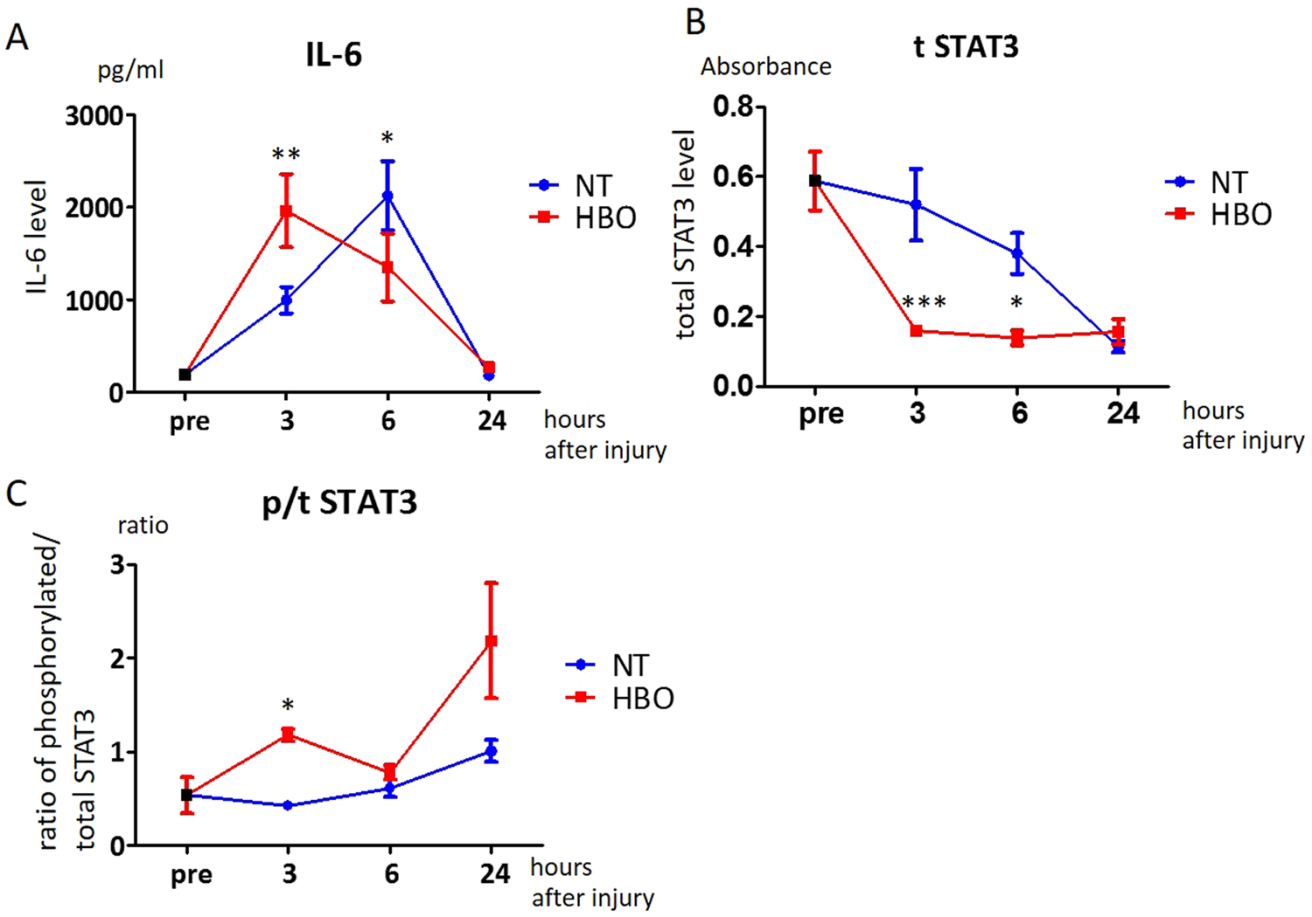
HBO activated the early IL-6/STAT3 pathway in injured muscles. (**A**) The levels of IL-6 in the injured muscles were higher at 3 hours and lower at 6 hours in the HBO group than in the NT group after injury (n = 6–8). **P < 0.01, *P < 0.05 using two-way ANOVA followed by Bonferroni post-tests. (**B**) The levels of total STAT3 in the HBO group were lower than those in the NT group at 3 and 6 hours after injury (n = 6). ***P < 0.001, *P < 0.05 using two-way ANOVA followed by Bonferroni post-tests. (**C**) The ratios of phosphorylated STAT3 to total (p/t) STAT3 were significantly higher in the HBO group than in the NT group at 3 hours after injury. *P < 0.05 using two-way ANOVA followed by Bonferroni post-tests. Data are the mean ± SEM.

We investigated whether the expression of IL-6 and STAT3 changed in injured muscle within 24 hours after injury. IL-6 expression immediately increased after injury and peaked at 3 hours in the HBO group (P < 0.01) (NT, 995 ± 144 pg/mg; HBO, 1964 ± 396 pg/mg) and 6 hours in the NT group (P < 0.05) (NT, 2126 ± 376 pg/mg; HBO, 1350 ± 368 pg/mg), and these increases were significant (Fig. 7A). Significantly lower levels of total STAT3 were present in the HBO group than in the NT group at 3 hours (P < 0.001) (NT, 0.52 ± 0.10; HBO, 0.16 ± 0.01) and 6 hours (P < 0.05) (NT, 0.38 ± 0.06; HBO, 0.14 ± 0.02) (Fig. 7B). The ratio of phosphorylated to total STAT3 was significantly higher at 3 hours in the HBO group than in the NT group (P < 0.05) (NT, 0.42 ± 0.05; HBO, 1.17 ± 0.07) (Fig. 7C). HBO thus induced IL-6 expression earlier than non-treatment. This resulted in activation of expression of the downstream protein STAT3 in the acute phase after injury.

## Discussion

In this study, we demonstrated that HBO dramatically reduced hypoxia and that the oxygenized environment lasted for 30 hours after even only one session of HBO. During HBO, the oxygen concentration increased in the later half of 2.5 ATA (Fig. 1A), possibly as a result of decreased blood circulation caused by arterial constriction under the high oxygen concentration to maintain homeostasis^13^. HBO reduced the volume of contused calf muscles in the acute phase after injury. HBO also activated the IL-6/STAT3 pathway, altered the amounts of circulating and accumulating macrophages, and promoted satellite cell proliferation and differentiation in the acute phase after injury, resulting in increased regeneration of muscle fibers and muscle strength (Figure S2).

Acute inflammation after skeletal muscle injury involves the production of inflammatory cytokines, recruitment of neutrophils, and increased vascular permeability^12,14,15^. Edema and tissue hypoxia occur as a result of increased extracellular fluid accumulation and local circulatory dysfunction caused by increased internal pressure^16,17^. Oxygen deprivation during hypoxia in the injured muscles causes a switch to anaerobic glycolysis for ATP production, which is only tolerable for a limited time. When the anaerobic pathway cannot produce ATP, membrane ion channel dysfunction and failure of cell homeostasis occur. This phenomenon leads to secondary skeletal muscle fiber necrosis^18,19^. Thus, oxygenation is expected to prevent secondary damage in injured muscles. We found that improvement of oxygenation in the injured environment by HBO reduced early hindlimb volume, extracellular fluid accumulation, and vascular permeability. These findings indicate that HBO prevented hypoxia-induced inflammation in the acute phase after muscle injury.

After muscle injury, monocytes circulating in blood vessels and bone marrow-derived monocytes specially induced and mobilized by the injury invade the injured tissue and differentiate into pro-inflammatory macrophages. Monocyte-derived macrophages differentiate into inflammatory M1 macrophages (M1) in the acute phase of inflammation. These M1 cells then differentiate into anti-inflammatory M2 macrophages (M2), which reduce local inflammation, contributing to myoblast fusion and myofiber regeneration by stimulating proliferation of satellite cells, which plays an essential role in muscle regeneration^20^. A recent study showed the presence of macrophages of different origins, i.e., circulating monocyte-derived macrophages and tissue-resident macrophages, which are considered to obtain proliferating capability^21,22^. Tissue-resident macrophages also play a significant role in recovery from muscle injury by proliferating to act as M2 cells that accelerate regeneration^23–25^. In the M2 population, CD206- (C-type mannose receptor 1) expressing cells are considered to play a main role in the resolution of inflammation because a lack of CD206 results in elevation of serum inflammatory protein levels. As several types of tissue-resident macrophages express CD206 in mice^26^, the CD206-positive cells observed at day 3 and 5 might represent an acute response. In contrast, CD163 has a high basal expression, amplified by IL-10 and glucocorticoids^27,28^, and it is thus possible that the delayed CD163 expression observed here reflected the anti-inflammatory or stress response against muscle injury. CD206-positive macrophages are also considered to produce hepatocyte growth factor in injured muscle, which may facilitate muscle fiber regeneration^29^. We found that in the macrophage response, macrophages labeled with CD68 accumulated 2 days earlier in the muscles of the HBO group compared to those of the NT group. The amount of M2 cells labeled with CD206 also increased earlier after injury in the HBO group than in the NT group (Fig. 5). Based on the difference in the time course of macrophage infiltration between the NT and HBO groups, HBO thus accelerated the transition from an inflammatory to an anti-inflammatory response and the subsequent regeneration process after skeletal muscle injury. This result means that HBO may exert both an anti-inflammatory effect and stimulation of muscle regeneration through enhanced differentiation of macrophages to the CD206-positive M2 phenotype.

Satellite cells express the transcription factor Pax7 in the quiescent state, and when they are activated, the myogenic regulatory factor MyoD is induced such that it is co-expressed with Pax7. During differentiation, Pax7 is downregulated, and MyoD stimulates further differentiation into the myoblast lineage. However, a minority of proliferating satellite cells returns to quiescence to prepare for the next injury^30^. In this study, HBO increased the number of proliferating satellite cells (both Pax7^+^/MyoD^+^ cells and Pax7^+^/Ki67^+^ cells) at 1 and 3 days; the number of differentiating satellite cells at 3 and 5 days; and the number of quiescent satellite cells at 3 and 5 days after injury (Fig. 6). The peak of satellite cell proliferation was accelerated after HBO. The increase in proliferating satellite cells resulted in an increased amount of quiescent and differentiating satellite cells. In drug-induced muscle injury, mRNA expression of MyoD was previously shown to increase at 1 and 3 days after injury^31^. Taken together, these results suggest that HBO enhances satellite cell proliferation and differentiation in the acute phase of muscle injury. Thus, HBO induces faster recovery from muscle contusion injury. Indeed, the twitch and tetanic muscle strength of HBO-treated rats was stronger than that of non-treated rats 7 days after injury.

IL-6 is a cytokine derived from macrophages, neutrophils, and satellite cells in damaged skeletal muscles. STAT3 is a downstream mediator of IL6, and the IL-6/STAT3 pathway induces the phosphorylation and transportation of STAT3 from the cytoplasm to the nucleus, which then increases the expression of genes essential for satellite cell proliferation and differentiation^32,33^. The activation of this pathway also induces macrophage migration in regenerating muscles^34^. In our study, the peak IL-6 level occurred earlier in the HBO group than in the NT group, and the ratio of phosphorylated to total STAT3 was increased in the HBO group at 3 hours. Activation of the IL-6 pathway depleted the total STAT3 amount at 3 and 6 hours after injury. As our assay only detected cytosolic STAT3, the decrease in total STAT3 likely reflected IL-6-induced transit of STAT3 from the cytosol to the nucleus^35^. Early activation of this pathway by HBO thus enhanced the proliferation and differentiation of satellite cells and macrophage infiltration in injured muscles at the acute phase, finally accelerating myofiber regeneration and muscle strength.

We identified the mechanisms underlying HBO-mediated healing of skeletal muscle injury in an experimental rat model. Although repeated HBOs resulted in only a limited difference in the recovery effect after injury, the observed acceleration of muscle regeneration is expected to be clinically important because it could enable early recovery from severe muscle injury in high-level athletes, thereby reducing the associated short-term and long-term morbidity. This study provides evidence to support the clinical indication of HBO for skeletal muscle injury.

## Material and Methods

### Animals and experimental procedures

All animal experiments were performed under approved protocols and in accordance with the recommendations of the Institutional Animal Care and Use Committee of Tokyo Medical and Dental University. Male 10-week-old Wistar rats weighing 250–300 g were kept in standard cages at a constant temperature and light/dark cycle of 12 hours each, with the light/dark hours changing automatically, and given water and food (MF; Oriental Yeast, Tokyo, Japan) ad libitum throughout the experimental period. The experiments were conducted mainly during the day from 6:00 AM to 6:00 PM, and the total number of rats used was approximately 400. Each experiment and its subsequent analysis were performed blinded. The animals were anesthetized with an intraperitoneal injection of chloral hydrate (280 mg/kg) or gas anesthesia (1.5% to 3% isoflurane, 1.5 L/min flow). Muscle contusion was induced by the modified mass-drop method^36^. First, the targeted hindlimb was fixed distally at the Achilles’ tendon and proximally at the gastrocnemius muscle using percutaneous needles. The calf was placed on silicon clay (Therapy Putty; AliMed, Massachusetts, US). A solid aluminum cylinder (640 g) was dropped from a distance of 250 mm onto the impactor (diameter 10 mm, hemisphere surface), which was placed on the belly of the medial calf of the rat. Abnormal behavior did not occur, and immediate recovery of locomotion was observed 24 hours after injury. Subsequently, rats were randomly assigned to either non-treatment (NT) or HBO treatment (HBO) after muscle contusion. Approximately 15 minutes after contusion injury, the rats in the HBO group were placed in a hyperbaric experimental chamber in which 100% oxygen was administered at 2.5 ATA pressure for 2 h, with 15 minutes for compression, 120 minutes of exposure at 2.5 ATA, and 15 minutes for decompression, under 100% oxygen. The compression and decompression speeds were 0.1 ATA/min^31^. The treatment was performed once a day for 5 consecutive days.

### Oxygen concentration measurement

The partial pressure of oxygen (pO_2_) in the calf muscles was measured using an electrode^37^. The rat was held on the platform immediately after injury. A needle-type oxygen electrode (POE-10N; Bio Research Centre, Nagoya, Japan) was calibrated with saline at 37 °C bubbled with air, and then inserted into the gastrocnemius muscle where the impactor was placed. The oxygen concentration was monitored in the experimental HBO chamber at 25 °C (n = 2), with compression and decompression at 0.1 ATA/min and 15 minutes each for compression, exposure, and decompression. The oxygen concentration was measured at each time point, i.e., pre-contusion and 30 minutes, 3 hours, 6 hours, 24 hours, and 30 hours after contusion (n = 5).

### Volume measurement of the calf

Rats in the NT and HBO groups were placed in plastic cages and underwent µCT scanning (inspeXio SMX-100CT; Shimadzu, Kyoto, Japan) under gas anesthesia without sacrifice at 6 hours and at 1, 3, 5, and 7 days after the injury. The first measurement was performed 6 hours after injury, and 3.5 hours after the initial HBO. Scan data were analyzed by software (Phantoms; Ratoc System Engineering, Tokyo, Japan) to calculate the lower limb volume (soleus and gastrocnemius) between 5 and 22 mm proximal to the calcaneus. The changes in lower limb volume over time were evaluated longitudinally in each rat, with each rat measured at every time point (n = 13–20).

### Measurement of calf muscle wet weight

The animals were sacrificed for calf muscle sampling at 6 hours and 1, 3, 5, and 7 days after injury. The first measurement was performed 6 hours after injury, and 3.5 hours after the initial HBO. The wet weight of the dissected muscles was measured quickly using an electric balance (n = 8). The isolated calf muscles (soleus and gastrocnemius) were frozen in liquid nitrogen-cooled 2-methylbutane. The muscles were stored at −80 °C until further analysis.

### Histology of the calf muscle

Transverse sections of the calf muscles (soleus and gastrocnemius) were cut at 20 µm using a cryostat (CM 300; Leica Japan, Tokyo, Japan) at 2, 4, 6, 8, 10, and 12 mm proximal from the end of the Achilles’ tendon, and stored at −30 °C. The sections were stained with hematoxylin and eosin (H&E), and the slides were evaluated under light microscopy. Microphotographs were taken with a digital camera (Olympus AX70; Olympus, Tokyo, Japan) attached to a microscope (Olympus BX51; Olympus). The captured images were processed and analyzed using image analysis software (ImageJ; National Institutes of Health, Bethesda, MD). At 1 day after contusion injury, we randomly chose 10 high-power fields (HPFs) from the H&E-stained sections and measured the extracellular space, which was defined as non-cytoplasmic area using ImageJ software^38^. The proportion of extracellular space was calculated as a percentage for each section at 2, 4, 6, 8, 10, and 12 mm proximal from the end of the Achilles’ tendon. (n = 5) In the evaluation of regenerated fiber, myofibers with centrally located nuclei were considered to be regenerating after injury. We chose the section with largest injured area in slices from 8, 10, and 12 mm proximal to the end of the tendon. The injured area was determined as the cell-invaded area. We randomly selected 10 HPFs from the injured area and counted the number of regenerating myofibers in the injured area (n = 5).

### Vascular permeability evaluation

Vascular permeability was assessed by measuring the muscular uptake of intravenously injected Evans Blue (EB) dye^39,40^. We prepared NT and HBO groups in addition to an intact group without injury. At 1 day after injury, and two hours before calf muscle (soleus and gastrocnemius) collection, 0.5% EB solution was injected systemically via the jugular vein at a dose of 5 ml/kg. The rats were then sacrificed and perfused with saline for 30 minutes. Dissected intact or injured calf muscles were homogenized in 1 ml of N-dimethylformamide and incubated for 24 hours at room temperature. The samples were centrifuged at 6,500 g for 10 minutes, and the supernatants were measured at 620 nm using a spectrophotometer (iMark microplate reader, Bio-Rad Laboratories, CA, USA) (n = 10).

### Measurement of muscle isometric tensile strength

Rats in the NT and HBO group were anesthetized at 5 and 7 days after contusion. To evaluate gastrocnemius muscle function, the isometric tensile strength produced by stimulating the common tibial nerve was measured with a transducer (TB-653T; Nihon Koden, Tokyo, Japan) and recorded with a sensor interface (Power lab; AD Instruments Japan, Nagoya, Japan) and software (Power lab software; AD Instruments Japan). The common tibial nerve was stimulated with an electrostimulator (Neuropack µ; Nihon Koden) at 1 Hz (twitch) or 50 Hz (tetanus) using a surface electrode (UL2-2020, Unique Medical Co., Ltd., Tokyo, Japan), and the maximum strength was recorded^31^. The stimulation pulse was the minimum voltage that visibly contracted the gastrocnemius muscle. Maximum twitch and tetanic isometric tensile strengths of the injured (If) and non-injured (Nf) legs were measured. The strength ratio of the injured muscle to the non-injured muscle (ratio of If to Nf) was calculated (n = 6).

### Cell staining and sorting via flow cytometry

Blood samples were collected by cardiac puncture from the left ventricle before and 3, 6, 24, 48, and 72 hours after injury. The samples were washed in PBS solution. The whole blood was lysed using lysis buffer (BD Pharm Lyse, BD Biosciences) according to the manufacturer’s protocol. The cells from the blood were suspended at a density of 5 × 10^5^ cells/mL and stained for 30 minutes on ice with anti-rat CD11b-PerCP–eFluor710 antibody (eBioscience, San Diego, CA, USA) and PerCP-e710 Mouse IgG2a Isotype Control (eBioscience, San Diego, CA, USA), or with anti-CD68-FITC antibody (Bio-Rad Laboratories, CA, USA) and Mouse IgG1 Negative Control antibody FITC (Bio-Rad Laboratories, CA, USA) for cell surface marker analysis. Flow cytometric analysis of the cell surface was performed using a triple-laser FACS Verse system (BD) (n = 5)^41,42^.

### Immunohistochemistry

Transverse sections of the calf muscles were immersed in blocking solution (5% normal goat serum in PBS with 0.5% Triton X-100) for 30 min and then incubated overnight at 4 °C with the primary antibody (CD68, mouse monoclonal antibody, Bio-Rad Laboratories, CA, USA; CD163, mouse monoclonal antibody, Bio-Rad Laboratories, CA, USA; CD206, rabbit polyclonal antibody, Abcam, Cambridge, UK; Pax7, rabbit polyclonal antibody, Abcam, Cambridge, UK; MyoD, mouse monoclonal antibody, Santa Cruz, CA, USA; Ki67, rabbit polyclonal antibody, Novus Biologicals, Littleton, CO, USA) diluted 1:100 in PBS. The sections were washed three times for 5 min each in PBS and then incubated with the secondary antibodies (goat anti-mouse IgG-Alexa Fluor 594, goat anti-rabbit IgG-Alexa Fluor 488; Life Technologies Japan, Tokyo, Japan) diluted 1:400 in PBS for 1 h. The sections were then washed three times for 5 min each in PBS. Finally, the sections were incubated with DAPI (Life Technologies) for 1 min, washed in PBS, and mounted in mounting solution (PermaFluor; Thermo Fisher Scientific Japan, Yokohama, Japan). The positively stained cells were counted in 10 HPFs using the same histological procedures described above (n = 5).

### ELISA

Calf muscles (soleus and gastrocnemius) were isolated from the hindlimbs before injury and at 3, 6, and 24 hours after injury. The samples were trimmed, frozen, and crushed using a cell crusher. The samples were homogenized in a 1,000 µl of lysis/extraction reagent (CelLytick; Sigma-Aldrich, St. Louis, Missouri) and centrifuged at 14,000 rpm for 10 min at 4 °C, and the supernatants were extracted for assay. The concentrations of IL-6, total STAT3, and phosphorylated STAT3 were measured using enzyme-linked immunoassay (ELISA) kits (R&D Systems, Minneapolis, Minnesota) according to each manufacturer’s protocols (n = 6–8)^43^.

### Statistical analysis

The data are presented as the mean ± the standard error of the mean (SEM). All analyses were two-sided, and the significance level was 5%. The statistical analyses were performed using Windows SPSS Version 23.0 (IBM Japan, Ltd., Tokyo, Japan). Data were analyzed for normality with the Shapiro-Wilk test and for homoscedasticity with Levene’s test. We investigated longitudinal effects, the effect of HBO treatment, and the interaction between longitudinal effects and treatment using two-way ANOVA, with oxygen concentration, volume of hindlimbs, wet weight, FACS, immunostaining, and ELISA data as the outcome measures. Vascular permeability was compared across three groups (intact, NT, and HBO) at 24 hours after injury by one-way ANOVA followed by Bonferroni post-tests based on the normal distribution and homoscedasticity of the data. Data with normal distribution and homoscedasticity were subjected to Student’s t-test for comparison of the number of regenerated fibers and the tensile strength of the gastrocnemius muscle between two groups. The extracellular space data, which had only a normal distribution and without homoscedasticity, were subjected to Welch’s t-test for comparison between two groups.

## Electronic supplementary material

Supplementary Information

## References

[1] Souza J, Gottfried C (2013). Muscle injury: Review of experimental models. J Electromyogr Kinesiol..

[2] Delos D, Maak TG, Rodeo SA (2013). Muscle injuries in athletes: enhancing recovery through scientific understanding and novel therapies. Sports Health..

[3] Tiidus PM (2015). Alternative treatments for muscle injury: massage, cryotherapy, and hyperbaric oxygen. Curr Rev Musculoskelet Med..

[4] Järvinen TA, Järvinen TL, Kääriäinen M, Kalimo H, Järvinen M (2005). Muscle injuries: biology and treatment. Am J Sports Med..

[5] Yagishita K, Oyaizu T, Aizawa J, Enomoto M (2017). The effects of hyperbaric oxygen therapy on reduction of edema and pain in athletes with ankle sprain in the acute phase: A pilot study. Sport Exerc Med Open J..

[6] Gill AL, Bell CN (2004). Hyperbaric oxygen: its uses, mechanisms of action and outcomes. Q J M..

[7] Grim PS, Gottlieb LJ, Boddie A, Batson E (1990). Hyperbaric oxygen therapy. JAMA.

[8] Asano T (2007). Hyperbaric oxygen induces basic fibroblast growth factor and hepatocyte growth factor expression, and enhances blood perfusion and muscle regeneration in mouse ischemic hind limbs. Circ J..

[9] Bajek S, Nikolic M, Soic-Vranic T, Arbanas J, Bajek G (2011). Effect of hyperbaric oxygen treatment on myogenic transcriptional factors in regenerating rat masseter muscle. Coll Antropol..

[10] Gregorevic P, Lynch GS, Williams DA (2000). Hyperbaric oxygen increases contractile function of regenerating rat skeletal muscle after myotoxic injury. J Appl Physiol..

[11] Skyhar MJ (1986). Hyperbaric oxygen reduces edema and necrosis of skeletal muscle in compartment syndromes associated with hemorrhagic hypotension. J Bone Joint Surg Am..

[12] Strauss MB (1983). Reduction of skeletal muscle necrosis using intermittent hyperbaric oxygen in a model compartment syndrome. J Bone and Joint Surg Am..

[13] Stirban A (2009). Functional changes in microcirculation during hyperbaric and normobaric oxygen therapy. Undersea Hyperb Med..

[14] Nourshargh S, Hordijk PL, Sixt M (2010). Breaching multiple barriers: leukocyte motility through venular walls and the interstitium. Nat Rev Mol Cell Biol..

[15] Pillon NJ, Bilan PJ, Fink LN, Klip A (2013). Cross-talk between skeletal muscle and immune cells: muscle-derived mediators and metabolic implications. Am J Physiol Endocrinol Metab..

[16] Järvinen TA, Järvinen M, Kalimo H (2014). Regeneration of injured skeletal muscle after the injury. Muscles Ligaments Tendons J..

[17] Tidball JG (2005). Inflammatory processes in muscle injury and repair. Am J Physiol Regul Integr Comp Physiol..

[18] Eltzschig HK, Carmeliet P (2011). Hypoxia and Inflammation. N Engl J Med..

[19] Merrick MA (2002). Secondary injury after musculoskeletal trauma: a review and update. J Athl Train..

[20] Arnold L (2007). Inflammatory monocytes recruited after skeletal muscle injury switch into anti-inflammatory macrophages to support myogenesis. J Exp Med..

[21] Italiani P, Boraschi D (2014). From Monocytes to M1/M2 Macrophages: Phenotypical vs. Functional Differentiation. Front Immunol..

[22] Sieweke MH, Allen JE (2013). Beyond stem cells: self-renewal of differentiated macrophages. Science.

[23] Tidball JG, Villalta SA (2010). Regulatory interactions between muscle and the immune system during muscle regeneration. Am J Physiol Regul Integr Comp Physiol..

[24] Ciciliot S, Schiaffino S (2010). Regeneration of Mammalian Skeletal Muscle: Basic Mechanisms and Clinical Implications. Curr Pharm Des..

[25] Sciorati C, Rigamonti E, Manfredi AA, Rovere-Querini P (2016). Cell death, clearance and immunity in the skeletal muscle. Cell Death Differ..

[26] Rőszer T. Understanding the Mysterious M2 Macrophage through Activation Markers and Effector Mechanisms. *Mediat Inflamm*. **16**. 10.1155/2015/816460 (2015). 10.1155/2015/816460 PMC4452191 26089604

[27] Villalta SA (2011). Interleukin-10 reduces the pathology of mdx muscular dystrophy by deactivating M1 macrophages and modulating macrophage phenotype. Hum Mol Genet..

[28] Ritter M (2000). The scavenger receptor CD163: regulation, promoter structure and genomic organization. Pathobiology.

[29] Sakaguchi S (2014). Implication of anti-inflammatory macrophages in regenerative motoneuritogenesis: promotion of myoblast migration and neural chemorepellent semaphorin 3A expression in injured muscle. INT J BIOCHEM CELL BIOL..

[30] Yusuf F, Brand-Saberi B (2012). Myogenesis and muscle regeneration. Histochem Cell Biol..

[31] Horie M (2014). Enhancement of satellite cell differentiation and functional recovery in injured skeletal muscle by hyperbaric oxygen treatment. J Appl Physiol..

[32] Munoz-Canoves P, Scheele C, Pedersen B, Serrano A (2013). Interleukin-6 myokine signaling in skeletal muscle: a double-edged sword?. FEBS journal.

[33] Hoene M, Runge H, Haring HU, Schleicher ED, Weigert C (2013). Interleukin-6 promotes myogenic differentiation of mouse skeletal muscle cells: role of the STAT3 pathway. Am J Physiol Cell Physiol.

[34] Zhang C (2013). Interleukin-6/Signal Transducer and Activator of Transcription 3(STAT3) Pathway Is Essential for Macrophage Infiltration and Myoblast Proliferation during Muscle Regeneration. J. BIOL. CHEM..

[35] Ndubuisi M, Guo G, Fried V, Etlinger J, Sehgal P (1999). Cellular Physiology of STAT3: Where’s the Cytoplasmic Monomer?. J. Biol. Chem..

[36] Kami K (1993). Changes of vinculin and extracellular matrix components following blunt trauma to rat skeletal muscle. Med Sci Sports Exerc..

[37] Yomura Y (2007). Direct, real-time, simultaneous monitoring of intravitreal nitric oxide and oxygen in endotoxin-induced uveitis in rabbits. Life Sci..

[38] Gejo R (2000). Magnetic Resonance Imaging and Histologic Evidence of Postoperative Back Muscle Injury in Rats. Spine..

[39] Humphrey DM (1993). Measurement of cutaneous microvascular exudates using Evans blue. Biotech Histochem..

[40] Wooddell CI (2010). Use of Evans blue dye to compare limb muscles in exercised young and old mdx mice. Muscle Nerve..

[41] Troidl C (2013). The temporal and spatial distribution of macrophage subpopulations during arteriogenesis. Curr Vasc Pharmacol..

[42] Repo H, Jansson SE, Leirisalo-Repo M (1993). Flow cytometric determination of CD11b upregulation *in vivo*. J Immunol Methods..

[43] Sakuma Y (2016). Muscle injury in rats induces upregulation of inflammatory cytokines in injured muscle and calcitonin gene-related peptide in dorsal root ganglia innervating the injured muscle. MUSCLE & NERVE..

