# Supplementary material for: Hyperbaric oxygen reduces inflammation, oxygenates injured muscle, and regenerates skeletal muscle via macrophage and satellite cell activation
Source: Sci Rep. 2018 Jan 22;8:1288. doi: 10.1038/s41598-018-19670-x (PMC5778072; doi:10.1038/s41598-018-19670-x)
Supplement: Supplementary file 1 — Supplementary Information [file 41598_2018_19670_MOESM1_ESM.pdf]

Supplementary Information

Title

Hyperbaric oxygen reduces inflammation, oxygenates injured muscle, and regenerates skeletal muscle via macrophage and satellite cell activation.

Authors and affiliations

Takuya Oyaizu [a, b], *Mitsuhiro Enomoto [b, c], Naoki Yamamoto [a, b], Kunikazu Tsuji [d], Masaki Horie [b], Takeshi Muneta [e], Ichiro Sekiya [f], Atsushi Okawa [a], Kazuyoshi Yagishita [b, c]

a Department of Orthopaedic Surgery, Tokyo Medical and Dental University, Bunkyo-ku, Tokyo 113-8519, Japan

b Hyperbaric Medical Center, Medical Hospital, Tokyo Medical and Dental University, Tokyo 113-8519, Japan

c Sports Medicine Clinical Center, Medical Hospital, Tokyo Medical and Dental University, Bunkyo-ku, Tokyo 113-8519, Japan

d Department of Cartilage Regeneration, Tokyo Medical and Dental University, Bunkyo-ku, Tokyo 113-8519, Japan

e Department of Joint Surgery and Sports Medicine, Bunkyo-ku, Tokyo 113-8519, Japan

f Center for Stem Cell and Regenerative Medicine, Bunkyo-ku, Tokyo 113-8519, Japan

Corresponding Authors

Mitsuhiro Enomoto, M.D., Ph.D.

Hyperbaric Medical Center and Sports Medicine Clinical Center, Medical Hospital, Tokyo Medical and Dental University

1-5-45, Yushima, Bunkyo-ku, Tokyo 113-8519, Japan

Phone: +81-3-5803-5279, Fax: +81-3-5803-5281

E-mail: enomorth@tmd.ac.jp

Fig.S1

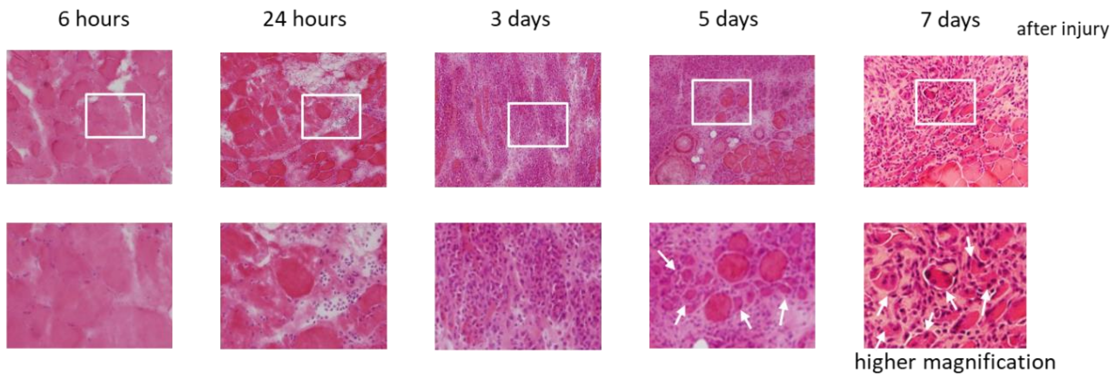

Figure S1. Histological changes of contused calf muscles.

The muscle fiber membrane was disrupted at 6 hours after contusion. Inflammatory cells were observed at 24 hours. Extensive aggregation of inflammatory cells occurred at 3 days. Newly regenerated myofibers with central nuclei (arrows) began to appear at 5 days and their numbers increased by 7 days after injury.

Fig.S2

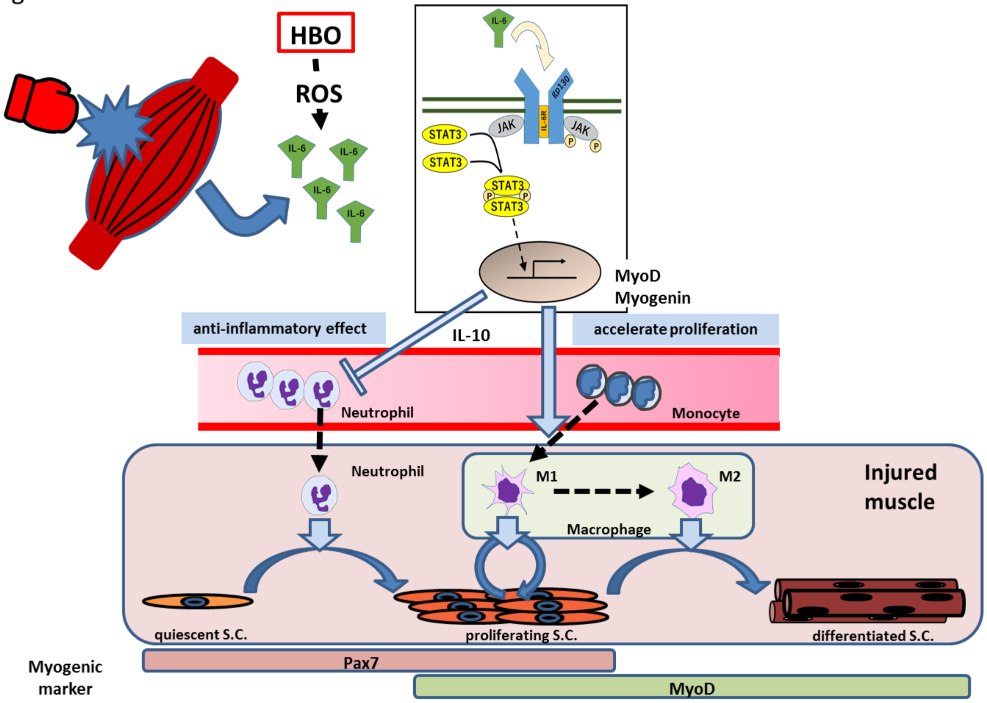

Figure S2. Mechanism underlying the effect of HBO on skeletal muscle regeneration.

Possible mechanism underlying the effect of HBO on skeletal muscle regeneration. HBO elevates the oxygen concentration of injured muscle, which induces reactive oxygen species (ROS). ROS production stimulates IL-6 secretion in the skeletal muscle. The IL-6/STAT3 pathway is stimulated transiently, which results in anti-inflammation via IL-10 and cell proliferation via MyoD and myogenin. Circulating neutrophils are suppressed, and monocyte recruitment to the injured muscle is accelerated. Macrophage invasion and M2 macrophage conversion are stimulated, resulting in satellite cell proliferation and myofiber regeneration.
